# Supplementary material for: Genome-wide association analyses highlight the role of the intestinal molecular environment in human gut microbiota variation
Source: Nat Genet. 2026 Feb 13;58(3):540–9. doi: 10.1038/s41588-026-02512-2 (PMC12987725; doi:10.1038/s41588-026-02512-2)
Supplement: Supplementary file 1 — Supplementary Note [file 41588_2026_2512_MOESM1_ESM.pdf]

nature genetics

Supplementary information https://doi.org/10.1038/s41588-026-02512-2

Genome-wide association analyses highlight the role of the intestinal molecular environment in human gut microbiota variation

In the format provided by the authors and unedited

GENOME-WIDE ASSOCIATION ANALYSES HIGHLIGHT THE ROLE OF THE INTESTINAL MOLECULAR ENVIRONMENT IN HUMAN GUT MICROBIOTA VARIATION

SUPPLEMENTARY NOTE

Malmö Diet and Cancer Study

The Malmö Diet and Cancer Study (MDC) is a prospective population-based cohort that enrolled 31,088 men and women aged 44-74 between 1991 and 1996.[1] A random subset of these participants was invited to a follow-up in 2007, when plasma levels of glucagon-like peptide-1 (GLP-1) were measured before and after an oral glucose load in participants without diabetes. MDC samples were genotyped at the Broad genotyping facility using the Infinium OmniExpressExome v1.0 B Beadchip array (Illumina) for MDC.[2] Genotype imputation for autosomal chromosomes was performed using the Haplotype Reference Consortium (HRC) r1.0.3 on the Michigan Server. Blood samples were analyzed for GLP-1 concentrations, including both the intact hormone and the GLP-1 9-36 amide metabolite, using a radioimmunological assay developed in-house (no. 89390; sensitivity <1 pmol/l).[2,3]

Prevalence, Prediction, and Prevention of type 2 diabetes-Botnia Study

The Prevalence, Prediction, and Prevention of type 2 diabetes (PPP)-Botnia Study is a population-based study that began in 2004.[4] Participants were randomly selected from the National Finnish Population Registry, representing 6-7% of the population aged 18-75. Of the original 5,208 participants, 3,850 (77%) attended the first follow-up in 2011-2015, where GLP-1 levels were measured.[5] Genotyping was performed on a FinnGen ThermoFisher Axiom custom array at the Thermo Fisher genotyping service facility in San Diego, CA, USA.[6] Standard quality control filters were applied to filter SNPs and samples before imputation. SNPs were excluded for monomorphism, low call rate, or Hardy-Weinberg deviation. Samples with duplications or low call rates, unexpected relatives, sex mismatches, heterozygosity outliers, and ancestral outliers (non-Europeans) were excluded. Genotype imputation was carried out using the population-specific SISu v3 reference panel with Beagle 4.1 (v.08Jun17.d8b, https://faculty.washington.edu/browning/beagle/b4_1.html).[7] Blood GLP-1 was measured using GLP-1 (total) radioimmunoassay (GLP1T-36HK, EMD Millipore) with high specificity to GLP-1 (GLP-2, glucagon, and exendin <0.2%). The range was 3–333 pmol/l.

Genotyping and imputation

SCAPIS

DNA extraction was performed at Karolinska Institutet Biobank (Stockholm, Sweden) and genotyped in 10 batches by SNP&SEQ Technology Platform (SciLifeLab, Uppsala, Sweden) using Illumina Infinium Global Screening Array Multiple Disease (GSA-MD) version 3 (Illumina, San Diego, CA, USA). Pre-imputation quality control included removing samples and markers with >2% missing data, samples from individuals of non-European ancestry, failure in sex check, heterozygosity measure >3 SD, and markers with Hardy-Weinberg equilibrium $P<1x10^{-8}$, minor allele frequency (MAF) <0.1%. Palindromic SNPs, i.e., A/T and G/C SNPs, were removed if minor allele frequency was >0.4 to reduce the risk of markers with allele switches. SNPs with other alleles than the corresponding SNP in the imputation reference panel (HRC r1.1) were also removed, together with SNPs with more than a 0.2 allele frequency difference between data and reference panel. The final imputation input dataset comprised 538,960 markers. The Sanger Imputation Service was then used to impute the SCAPIS genotype data to the HRC r1.1 with the pipeline "Pre-phasing and imputation with EAGLE2+PBWT".[8-10]

SIMPLER

For SIMPLER-V, DNA extraction and genotyping were performed at Eurofins Genomics (Ebersberg, Germany) using the Illumina Infinium GSA version 3 (Illumina, San Diego, CA, USA). For SIMPLER-U, DNA extraction was done at the Karolinska Institutet Biobank and genotyping was performed by the SNP&SEQ Technology Platform using the Illumina Infinium GSA-MD v1 (Illumina, San Diego, CA, USA). Pre-imputation quality control included removing samples and markers with >2% missing data, samples from individuals of non-European ancestry, failure in sex check, heterozygosity measure >3 SD, and markers with Hardy-Weinberg equilibrium $P<1x10^{-7}$, minor allele count <20 counts. Before imputation, genetic variants with different allele frequencies than the HRC r1.1 (GRCh37) reference panel (>0.15 for SIMPLER-V or >0.2 for SIMPLER-U) were also removed. Imputation to the HRC r1.1 panels took place at the Michigan Imputation Server using EAGLE v2.4 + minimac v4.[11]

MOS

The DNA extraction of MOS blood samples was carried out using the QIAamp DNA Blood Maxi Kit (Qiagen, Hilden, Germany). The genotyping of MOS samples was performed by Erasmus MC Genomics Core Facility (Rotterdam, the Netherlands) in two batches. The first batch was

genotyped in 2017 using Illumina GSA-MD v1 and the second in 2020 using GSA-MD v3. Pre-imputation quality control criteria and imputation procedures were identical to SCAPIS.

HUNT

DNA extracted from blood samples in HUNT and was genotyped using one of four Illumina HumanCoreExome arrays.[12] The genotyping process used the GenTrain v2.0 algorithm in GenomeStudio v2011.1. Samples were excluded if the call rate was <99%, large chromosomal copy number variants were detected, contamination exceeding 2.5% as estimated with BAF Regress was detected, or failure in sex check. Additionally, genetic variants with Hardy-Weinberg equilibrium $P<1\times10^{-4}$ were excluded. The samples were phased using Eagle2 v2.0.5, and all variants were subsequently imputed from the HRC r1.1 reference panel with the positional Burrows-Wheeler transform v3.1.

Validation of genotypes using Sanger sequencing

Direct genotyping using Sanger sequencing was performed to confirm the variants in rs10836441 (*OR51E1/2* locus) and rs4556017 (*MUC12* locus). For each of the two SNPs, 75 samples from SCAPIS and SIMPLER-V were selected (25 samples with imputed dosages of 0-0.5, 25 with 0.5-1.5, and 25 with 1.5-2.0). In total, 300 samples were selected for genotyping (2 SNPs x 2 cohorts x 75 samples). Four SCAPIS samples (two from each SNP) were unavailable in the biobank. Sanger sequencing was performed in forward and reverse mode using the primers: F 5´-CTCTGGGTCCTCTTTTCATCC-3´ and R 5´-TGAAATATCCCTATTTGTACA 3´ for rs10836441, and F 5´- TGATGTGTAAACCAGACAATA-3´ and R 5´- TCAAGGTGCTCAGGCATCAA- 3´ for rs4556017. PCR was performed at an annealing temperature of 50°C and 60°C for rs10836441 and rs4556017, respectively. Cleanup and Sanger sequencing were performed using ExoSAP-IT™ and the BigDye™ Terminator v1.1 Cycle Sequencing Kit, reducing the reaction volumes (Applied Biosystem, Waltham, MA, USA). Dye terminator removal was performed using an EdgeBio column plate (Edge Biosystem Inc., CA, USA), and the capillary electrophoresis was performed on a SeqStudio™ Genetic analyzer (Applied Biosystem).

The raw sequencing data were analyzed in Sequencher[13] v5.4.6 (Gene Codes) by an analyst blinded to the imputed data. Reads were trimmed, reverse reads reversed and complemented, and finally aligned using default settings in Sequencher. This software was also used to call the two SNPs. For rs10836441, two reverse reads were discarded due to unsuccessful sequencing. Eleven samples had an initial mismatch of the SNP calls from the forward and

reverse reads; however, manual inspection of chromatograms revealed that the forward reads signal quality was excellent, while the reverse reads contained capillary migration artefacts with stretched peaks. Thus, these were called according to the forward read (all heterozygotes). For the remaining 135 samples, the forward and reverse SNP calls agreed. For rs4556017, two forward reads and two reverse reads were discarded due to unsuccessful sequencing, but the complementary read held high quality. For the remaining 144 samples, forward and reverse calls agreed. To evaluate the concordance between imputed genotype dosages and the Sanger sequencing genotypes, we assigned a predicted genotype with value 0 for dosages 0-0.49, 1 to dosages 0.5-1.49, and 2 to dosages 1.5-2.

Colocalization

Pairwise colocalization analyses were performed to investigate whether microbial richness and the eight study-wide significant species colocalize at the identified study-wide significant loci and with the following traits: secondary bile acids in SCAPIS using Metabolon metabolomics data[14] as described below, sex hormone-binding globulin (UK Biobank),[15] waist-to-hip ratio adjusted for body mass index (WHRadjBMI) (GIANT + UK Biobank),[16] low-density lipoprotein (LDL) cholesterol (European ancestry results of trans-ancestry meta-analysis),[17] inflammatory bowel disease (IBD) (European ancestry meta-analysis),[18] glucose (UK Biobank, Neale v2 - http://www.nealelab.is/uk-biobank/), and stool frequency (a subset of UK Biobank + four smaller studies).[19] GWAS summary statistics were harmonized using the harmonize_data function of the TwoSampleMR v0.5.7 R package (R v4.3.1), and colocalization was assessed using the coloc.abf function of the coloc v5.2.2 R package. All variants in a window of 1Mb surrounding the lead SNP of a loci were used as input. For multiallelic variants, the allele pair with the lowest *P*-value of the GWAS of "trait 1", which was the GWAS of the trait associated with the investigated locus, was used as input. coloc requires the number of samples as input, and if the number of analyzed samples was different for variants in a GWAS, the median was used instead.

Mendelian randomization

We performed two-sample Mendelian randomization (MR) analyses to investigate bidirectional effects between specific species (*Clostridium saudiense, Turicibacter sanguinis, Intestinibacter* sp9005540355) and BMI, WHR, and LDL cholesterol. The genetic associations with BMI and WHRadjBMI were obtained from a meta-analysis of GWAS in GIANT and UK Biobank data (max *n*=694,649).[20,21] Genetic associations with LDL cholesterol were obtained

from a GWAS of European ancestry (*n*=1.32M).[17] Genetic instruments were created by LD-clumping (LD R2 <0.001 within 10 Mb) of genetic variants associated with a species or trait with a $P<5x10^{-8}$. LD-clumping was performed using the European 1000 Genomes reference.[22] MR analyses were performed when genetic instruments comprised >1 variant. For *Clostridium saudiense* and *Turicibacter sanguinis,* the instruments consisted of two loci, and for *Intestinibacter* sp9005540355 of four loci. Sensitivity analyses were performed when the species were the exposure using $P<5x10^{-6}$ to create genetic instruments. Inverse-weighted MR, weighted-median MR, and MR-Egger analyses were conducted using the R package MendelianRandomization v0.9.0 (R v4.2.2). As per default in this package, inverse-weighted MR with fixed-effects was used for genetic instruments with two variants and multiplicative random-effects with >2 variants. Multiple testing was addressed using Benjamini-Hochberg method considering a false discovery rate (FDR) of 5% and the significance was expressed as q-values.

CHAMP^{TM} Profiler

Microbial taxonomic annotation was performed at Cmbio (Copenhagen, Denmark) using the CHAMP^{TM} profiler based on the Human Microbiome Reference HMR05 catalog.[23] The HMR05 catalog is based on 30,482 samples from 9 human body sites, including prokaryotic metagenome-assembled genomes (MAGs) mainly from the Unified Human Gastrointestinal Genome collection,[24] the Early-Life Gut Genomes catalog,[25] and selected genomes from the National Center for Biotechnology Information (NCBI) and Pathosystems Resource Integration Center (PATRIC). MAGs were clustered by species using the Genome Taxonomy Database Toolkit (GTDB-Tk release R214),[26] while unannotated MAGs were clustered at 95% identity using FastANI. The catalog includes 6,809 microorganisms, of which >4,700 were found in our studies. Eukaryotic species were identified from various sources, including a public list of pathogens and gut fungal species from the Human Microbiome Project.[27,28] Protein-coding genes were clustered to create a nonredundant gene catalog, and up to 250 signature genes were identified for each species/subspecies.

A gene count table was created using the number of uniquely mapped read pairs for each gene. The relative abundance of each species (MAGs) was calculated based on the species signature genes with observed read counts within the expected 99% quantile and normalized sample-wise so that the total abundance of all species summed to 100%. The expected 99% quantile of read counts was calculated for each gene based on a negative binomial distribution with a mean proportional to the effective gene length and dispersion as $\log_2$(effective gene length).

For functional annotation, catalogue genes were annotated to the gut modules (gut metabolic modules v1.07 and gut brain modules v1.0) using EggNOG-mapper[29] v2.0.1. Module abundances were defined as the sum of the relative abundances of all species containing that module. We removed 34 gut brain modules with relative abundance r>0.95 with gut metabolic modules.

External replication

Associations passing the study-wide threshold were assessed in HUNT by applying the same models as in the Swedish cohorts and using REGENIE[30] with the same model specifications. We further assessed the validity of our findings using summary statistics from the published FINRISK[31] and Dutch Microbiome Project[32] studies. The FINRISK study included species with >25% prevalence, applied centered log-ratio transformation to relative abundances and analyzed genetic associations in a linear mixed model (BOLT-LMM v.2.3.2). The Dutch Microbiome Project study focused on taxa with mean relative abundance >0.001% across all samples and present in ≥1,000 of the 7,738 individuals (~13% of population), treating all zero values as missing data, log-transformed relative abundances followed by a rank-based inverse-normal (RIN) transformation, and analyzed genetic associations using a linear mixed model implemented in SAIGE v.0.38. We identified the best-matching species to our study-wide associated species using the Taxon History in the GTDB database (https://gtdb.ecogenomic.org/taxon-history). The study in FINRISK used an earlier GTDB version (R89) for taxonomic annotations, while the Dutch study annotated their taxa using MetaPhlAn2, which uses NCBI nomenclature. Public GWAS results for the matched species were accessed, and beta, standard error, P, and species prevalence were extracted for the relevant SNPs. If the SNPs were unavailable, we used proxies with $r2>0.8$ and aligned the alleles.

Plasma Metabolomics

Fasting plasma samples from SCAPIS were analyzed by Metabolon Inc. (TX, USA) using ultrahigh-performance liquid chromatography-tandem mass spectroscopy in four parallel processes to optimize metabolite identification. Metabolites were annotated using Metabolon Inc.'s internal reference library based on retention time, mass-to-charge ratio, and chromatography. To investigate the association of "study-wide-associated" species with plasma metabolite levels, we conducted partial Spearman's correlations adjusted for age, sex, place of birth, and metabolomics delivery batch using the ppcor v1.1 R package (R v4.3.1). Metabolites

detected in less than 100 samples were removed. Metabolites detected in less than 2% of the samples and drug metabolites were converted to a binary (detected, not detected) variable, except for drug metabolites 2,6-dihydroxybenzoic acid and salicylate, which were detected in more than 99% of the individuals. Metabolon deposited spectral data from the first analytical stage (MS1) for 125 anonymized samples from SCAPIS-Uppsala in MetaboLights under accession number MTBLS407 (https://www.ebi.ac.uk/metabolights/MTBLS407).

Short-chain fatty acids

^{12}C-derivatized samples were prepared and mixed with custom-synthesized ^{13}C-labelled internal standards (for each short-chain fatty acid) and analyzed in 6500+ QTRAP triple-quadrupole MS, equipped with an atmospheric pressure chemical ionization source in the negative-ion mode. Chromatographic separations were performed on Phenomenex Kinetix Core-Shell C18 UPLC with Security Guard ULTRA Cartridges.

Simulations

We conducted simulations to assess the type I error of logistic and linear models, identifying the species prevalence cut-off where a linear model becomes unreliable. For a 50% prevalence example, we selected a real species distribution from the SIMPLER-U (the smallest study in the meta-analysis) with 49-51% prevalence. We then performed sampling with replacement to generate a sample size of 981, followed by RIN transformation of values. The exposure was independently simulated using a multinomial distribution with a MAF of 0.05, assuming Hardy-Weinberg equilibrium. Univariate linear regression models were run to extract *P*, with 10 billion simulations determining type I error at thresholds below 5×10^{-8}. For logistic regressions at 5% and 10% prevalence thresholds, with and without Firth correction, we ran 100 million simulations. The outcome was species presence (yes/no), and the exposure was generated similarly. Regular logistic regression showed some type I error inflation, which was not observed with the Firth correction. Given these simulations using the smallest sub-cohort and lowest MAF, we believe our approach to the type I error issue is safe and conservative. Simulations were carried out in R v4.1.1.

Supplementary Note References

1. Manjer, J. *et al.* The Malmo Diet and Cancer Study: representativity, cancer incidence and mortality in participants and non-participants. *Eur J Cancer Prev* **10**, 489-99 (2001).

2. Almgren, P. *et al.* Genetic determinants of circulating GIP and GLP-1 concentrations. *JCI Insight* **2**, e93306 (2017).
3. Lindgren, O. *et al.* Incretin hormone and insulin responses to oral versus intravenous lipid administration in humans. *J Clin Endocrinol Metab* **96**, 2519-24 (2011).
4. Pyykkonen, A.J. *et al.* Stressful life events and the metabolic syndrome: the prevalence, prediction and prevention of diabetes (PPP)-Botnia Study. *Diabetes Care* **33**, 378-84 (2010).
5. Isomaa, B. *et al.* A family history of diabetes is associated with reduced physical fitness in the Prevalence, Prediction and Prevention of Diabetes (PPP)-Botnia study. *Diabetologia* **53**, 1709-13 (2010).
6. Kurki, M.I. *et al.* FinnGen provides genetic insights from a well-phenotyped isolated population. *Nature* **613**, 508-518 (2023).
7. Browning, B.L. & Browning, S.R. Genotype imputation with millions of reference samples. *Am J Hum Genet* **98**, 116-26 (2016).
8. McCarthy, S. *et al.* A reference panel of 64,976 haplotypes for genotype imputation. *Nat Genet* **48**, 1279-83 (2016).
9. Loh, P.R. *et al.* Reference-based phasing using the Haplotype Reference Consortium panel. *Nat Genet* **48**, 1443-1448 (2016).
10. Durbin, R. Efficient haplotype matching and storage using the positional Burrows-Wheeler transform (PBWT). *Bioinformatics* **30**, 1266-72 (2014).
11. Larsson, S.C., Michaelsson, K., Mola-Caminal, M., Hoijer, J. & Mantzoros, C.S. Genome-wide association and Mendelian randomization study of fibroblast growth factor 21 reveals causal associations with hyperlipidemia and possibly NASH. *Metabolism* **137**, 155329 (2022).
12. Brumpton, B.M. *et al.* The HUNT study: A population-based cohort for genetic research. *Cell Genom* **2**, 100193 (2022).
13. Sequencher(R) DNA sequence analysis software. 5.4.6 edn (Gene Codes Corporation, Ann Arbor, MI, USA).
14. Dekkers, K.F. *et al.* An online atlas of human plasma metabolite signatures of gut microbiome composition. *Nat Commun* **13**, 5370 (2022).
15. Ruth, K.S. *et al.* Using human genetics to understand the disease impacts of testosterone in men and women. *Nat Med* **26**, 252-258 (2020).
16. Yengo, L. *et al.* Meta-analysis of genome-wide association studies for height and body mass index in ~700000 individuals of European ancestry. *Hum Mol Genet* **27**, 3641-3649 (2018).
17. Graham, S.E. *et al.* The power of genetic diversity in genome-wide association studies of lipids. *Nature* **600**, 675-679 (2021).
18. Liu, J.Z. *et al.* Association analyses identify 38 susceptibility loci for inflammatory bowel disease and highlight shared genetic risk across populations. *Nat Genet* **47**, 979-986 (2015).
19. Bonfiglio, F. *et al.* GWAS of stool frequency provides insights into gastrointestinal motility and irritable bowel syndrome. *Cell Genom* **1**, 100069 (2021).
20. Barton, A.R., Sherman, M.A., Mukamel, R.E. & Loh, P.R. Whole-exome imputation within UK Biobank powers rare coding variant association and fine-mapping analyses. *Nat Genet* **53**, 1260-1269 (2021).
21. Pulit, S.L. *et al.* Meta-analysis of genome-wide association studies for body fat distribution in 694 649 individuals of European ancestry. *Hum Mol Genet* **28**, 166-174 (2019).
22. Auton, A. *et al.* A global reference for human genetic variation. *Nature* **526**, 68-74 (2015).

23. Pita, S. *et al.* CHAMP delivers accurate taxonomic profiles of the prokaryotes, eukaryotes, and bacteriophages in the human microbiome. *Frontiers in Microbiology* **15**(2024).
24. Almeida, A. *et al.* A unified catalog of 204,938 reference genomes from the human gut microbiome. *Nat Biotechnol* **39**, 105-114 (2021).
25. Zeng, S. *et al.* A compendium of 32,277 metagenome-assembled genomes and over 80 million genes from the early-life human gut microbiome. *Nat Commun* **13**, 5139 (2022).
26. Chaumeil, P.A., Mussig, A.J., Hugenholtz, P. & Parks, D.H. GTDB-Tk: a toolkit to classify genomes with the Genome Taxonomy Database. *Bioinformatics* **36**, 1925-7 (2019).
27. Human Microbiome Project Consortium. Structure, function and diversity of the healthy human microbiome. *Nature* **486**, 207-14 (2012).
28. Human Microbiome Project Consortium. A framework for human microbiome research. *Nature* **486**, 215-21 (2012).
29. Cantalapiedra, C.P., Hernández-Plaza, A., Letunic, I., Bork, P. & Huerta-Cepas, J. eggNOG-mapper v2: functional annotation, orthology assignments, and domain prediction at the metagenomic scale. *Mol Biol Evol* **38**, 5825-5829 (2021).
30. Mbatchou, J. *et al.* Computationally efficient whole-genome regression for quantitative and binary traits. *Nat Genet* **53**, 1097-1103 (2021).
31. Qin, Y. *et al.* Combined effects of host genetics and diet on human gut microbiota and incident disease in a single population cohort. *Nat Genet* **54**, 134-142 (2022).
32. Lopera-Maya, E.A. *et al.* Effect of host genetics on the gut microbiome in 7,738 participants of the Dutch Microbiome Project. *Nat Genet* **54**, 143-151 (2022).
