# Supplementary material for: Genome-wide association analyses highlight the role of the intestinal molecular environment in human gut microbiota variation
Source: Nat Genet. 2026 Feb 13;58(3):540–9. doi: 10.1038/s41588-026-02512-2 (PMC12987725; doi:10.1038/s41588-026-02512-2)
Supplement: Supplementary file 2 — Reporting Summary [file 41588_2026_2512_MOESM2_ESM.pdf]

nature portfolio

Corresponding author(s): Tove Fall

Last updated by author(s): Sep 11, 2025

Reporting Summary

Nature Portfolio wishes to improve the reproducibility of the work that we publish. This form provides structure for consistency and transparency in reporting. For further information on Nature Portfolio policies, see our Editorial Policies and the Editorial Policy Checklist.

Statistics

For all statistical analyses, confirm that the following items are present in the figure legend, table legend, main text, or Methods section.

n/a	Confirmed	
☐	☒	The exact sample size (*n*) for each experimental group/condition, given as a discrete number and unit of measurement
☐	☒	A statement on whether measurements were taken from distinct samples or whether the same sample was measured repeatedly
☐	☒	The statistical test(s) used AND whether they are one- or two-sided<br>*Only common tests should be described solely by name; describe more complex techniques in the Methods section.*
☐	☒	A description of all covariates tested
☐	☒	A description of any assumptions or corrections, such as tests of normality and adjustment for multiple comparisons
☐	☒	A full description of the statistical parameters including central tendency (e.g. means) or other basic estimates (e.g. regression coefficient) AND variation (e.g. standard deviation) or associated estimates of uncertainty (e.g. confidence intervals)
☐	☒	For null hypothesis testing, the test statistic (e.g. *F*, *t*, *r*) with confidence intervals, effect sizes, degrees of freedom and *P* value noted<br>*Give P values as exact values whenever suitable.*
☒	☐	For Bayesian analysis, information on the choice of priors and Markov chain Monte Carlo settings
☒	☐	For hierarchical and complex designs, identification of the appropriate level for tests and full reporting of outcomes
☐	☒	Estimates of effect sizes (e.g. Cohen's *d*, Pearson's *r*), indicating how they were calculated

Our web collection on statistics for biologists contains articles on many of the points above.

Software and code

Policy information about availability of computer code

Data collection

No software was used for data collection.

Data analysis

All statistical analyses used R version 4.3.1, unless stated otherwise.

1. Bioinformatic processing, calculation of relative abundances, and microbial taxonomic annotation were conducted at Cmbio using the CHAMP profiler based on the Human Microbiome Reference HMR05 catalog.
2. Calculation of alpha diversity measures using vegan package v2.5-7
3. For simulations to assess the type I error of logistic and linear models, identifying the species prevalence cut-off where a linear model becomes unreliable (R v.4.1.1).
4. Principal components were calculated in the unrelated samples set with PLINK 1.9.
5. For imputation of the SCAPIS and MOS genotype data to the HRC r1.1, we used the Sanger Imputation Service with the pipeline "Pre-phasing and imputation with EAGLE2+PBWT". For the imputation of the SIMPLER -V and SIMPLER-U genotype data to the HRC r1.1 panel, the Michigan Imputation Server was used (EAGLE v.2.4 + minimac v4).
6. For genome-wide association analyses GWAS of microbiome composition (species and higher taxonomic levels) and function, metabolites, short-chain fatty acids, we used the software REGENIE v3.3, METAL v2011-03-25, PLINK v2.00-alpha-5-20230923, SumHer v6 and the meta v6.5-0 R package. SNPTEST v.2.5.6 was used for genome-wide association analyses of GLP-1.
7. For functional pathways mapping, we used FUMA v1.5.2.
8. For causal inferences analyses (Mendelian randomization), we used MendelianRandomization v0.9.0 R package (R v4.2.2).
9. For the stool metabolomics analyses coordinates were lifted over using Ensembl Variation 112 and https://genome.ucsc.edu/cgi-bin/hgLiftOver.
10. Colocalization was performed using TwoSampleMR v0.5.7 and coloc v5.2.2 R packages.

March 2021

11. Antigen - secretor status interactions were performed using the lmerTest v3.1-3 and metafor v4.4-0 R packages.
12. For the species - metabolite Spearman correlations we used the ppcor v1.1 R package.
13. For calculation of kinship estimator, we used KING as implemented in PLINK v.2.0.
14. For analyzing data from Sanger sequencing we used Sequencher v5.4.6
15. For functional annotation, EggNOG-mapper v.2.0.1 was used

Code related to the analyses in this study are available at https://github.com/MolEpicUU/GWAS_scripts and https://github.com/MolEpicUU/GWAS_microbiome and in Zenodo: https://doi.org/10.5281/zenodo.16947117 and https://doi.org/10.5281/zenodo.16925644

For manuscripts utilizing custom algorithms or software that are central to the research but not yet described in published literature, software must be made available to editors and reviewers. We strongly encourage code deposition in a community repository (e.g. GitHub). See the Nature Portfolio guidelines for submitting code & software for further information.

Data

Policy information about availability of data

All manuscripts must include a data availability statement. This statement should provide the following information, where applicable:

- Accession codes, unique identifiers, or web links for publicly available datasets
- A description of any restrictions on data availability
- For clinical datasets or third party data, please ensure that the statement adheres to our policy

Complete GWAS summary statistics are available in the GWAS catalog with accession numbers GCST90670368 to GCST90671939. De-hosted anonymized metagenomic sequencing data from SCAPIS used in this study can be found at the European Nucleotide Archive under accession number PRJEB51353. Single-cell RNA-seq data is available in the GEO repository with accession number GSE284419 and GSE269778, and on Dryad (https://doi.org/10.5061/dryad.8pk0p2ns8). The metagenomics, metabolomics and genetic data supporting the conclusions of this article were provided by the SCAPIS, SIMPLER, and MOS central data offices, and are not shared publicly due to confidentiality and ethical restrictions. Data will be shared by the respective data offices only after permission from the Swedish Ethical Review Authority (https://etikprovningsmyndigheten.se) and from the respective boards (https://www.scapis.org/data-access, https://www.simpler4health.se, and https://www.malmo-kohorter.lu.se/malmo-offspring-study-mos).

Human research participants

Policy information about studies involving human research participants and Sex and Gender in Research.

Reporting on sex and gender

The genome-wide association study performed here utilized genetic information and fecal metagenomic data from both sexes from 16,017 adults of European ancestry from four Swedish cohorts. The sex balance in the dataset is SCAPIS (52.5% female), SIMPLER-Västmanland (37.7% female) and SIMPLER-Uppsala (100% female), MOS (47.1% female).

SCAPIS and MOS: sex was obtained from the Swedish population register.
SIMPLER-Västmanland and SIMPLER-Uppsala: Invitation sent to all women for mammography screening (identified from the Swedish population register); men were identified from the Swedish population register.

Sex-stratified analyses were conducted for associations of study-wide significant loci-species combinations, and Mendelian randomization analyses of specific gut microbial species with several adiposity traits and LDL cholesterol.

Population characteristics

SCAPIS
The Swedish CArdioPulmonary BioImage Study (SCAPIS) is a multi-center cohort comprising 30,154 individuals aged 50-65. For this analysis, 8,733 participants of European ancestry from the Malmö and Uppsala sites with both gut microbiome and genotype data were included. At baseline, participants provided blood samples during the first visit and were asked to collect fecal samples at home, storing them at -20°C until samples were brought to the study center for the second visit. DNA extracted from whole blood was used for genotyping. Birth year and sex were obtained from the Swedish population register.

SIMPLER-Västmanland and SIMPLER-Uppsala
The Swedish Infrastructure for Medical Population-Based Life-Course and Environmental Research (SIMPLER; https://www.simpler4health.se/w/sh/en) includes data from two large, ongoing population-based studies: the Cohort of Swedish Men (COSM) and the Swedish Mammography Cohort (SMC).58 The COSM initially enrolled 48,850 men born between 1918 and 1952 living in Västmanland and Örebro counties in 1997. The SMC enrolled 66,651 women by sending invitations to all women born between 1914 and 1948 living in Uppsala and Västmanland counties between 1987 and 1990. The current analysis is based on a randomly selected subsample from these studies who were invited for clinical examination with genotype and gut microbiome data: SIMPLER-Västmanland (SIMPLER-V) and SIMPLER-Uppsala (SIMPLER-U). SIMPLER-V includes 4,515 COSM and SMC participants from Västmanland examined between 2010 and 2019. SIMPLER-U includes 981 women from the county of Uppsala, examined between 2003 and 2009 (no stool collected) and re-examined between 2015 and 2019 (stool collected).

MOS
The Malmö Offspring Study (MOS) includes participants aged ≥18 who are children or grandchildren of participants from the Malmö Diet and Cancer Study (MDC)-Cardiovascular Cohort, a subset of the larger MDC.59 Data collection in MOS began in 2013 and included 4,721 participants by 2020. The current study included 1,788 participants with genotype and gut microbiome data who attended baseline measurements between 2013 and 2017.

Recruitment

SCAPIS: 30,154 participants aged 50-64 years invited from a random selection from the Swedish population register in areas adjacent to study sites.

SIMPLER-Västmanland and SIMPLER-Uppsala: (SMC) From March 1987 to December 1990, all women living in Uppsala County of central Sweden and who were born in 1914 through 1948 (n = 48,517) and all women living in the adjacent Västmanland County (n = 41,786) who were born in 1917 through 1948 received an invitation by mail to participate in a population-based mammography screening program, along with a questionnaire. Returning of the questionnaire was their informed consent. The SMC population is comparable to the general Swedish population with regards to age distribution, education level and body mass index (BMI). (COSM) In the fall of 1997, all men born in 1918 through 1952 living in Västmanland and Örebro counties in central Sweden (n = 100,303) received an invitation to participate in the study, along with a self-administered questionnaire. The COSM population is comparable to the general Swedish population with regards to age distribution, education level and BMI.

MOS: Participants were children and grandchildren of index individuals in Malmö Diet and Cancer Study—Cardiovascular Cohort, which was a random, subpopulation of the Malmö Diet and Cancer Study. The participants were 18 years or older and living in Malmö or the nearby catchment area.

Ethics oversight

The current association study has been approved by the Swedish Ethical Review Authority (DNR 2022-06137-01 and DNR 2024-01992-02). All participants in the respective cohorts below have provided written informed consent to participate in the studies and have their samples and data collected, stored, and processed. The Swedish Ethical Review Board has approved the data collection, and the approval numbers are provided: SCAPIS (DNR 2010-228-31M), SIMPLER (DNR 2009/2066-32, DNR 2009/1935-32, DNR 2010/0148-32, DNR 2014/892-31/3), MDC (DNR 532/2006, DNR 51-90), and MOS (DNR 2012-594). The PPP-Botnia study received approval from the Ethics Committee of Helsinki University (approval number 608/2003). The HUNT study was approved by the local ethical review board (Regionale kommitter for medicinsk og helsefaglig forskningsetik Midt-Norge; REK-656785).

Note that full information on the approval of the study protocol must also be provided in the manuscript.

Field-specific reporting

Please select the one below that is the best fit for your research. If you are not sure, read the appropriate sections before making your selection.

☒ Life sciences ☐ Behavioural & social sciences ☐ Ecological, evolutionary & environmental sciences

For a reference copy of the document with all sections, see nature.com/documents/nr-reporting-summary-flat.pdf

Life sciences study design

All studies must disclose on these points even when the disclosure is negative.

Sample size

The sample size was based on the number of participants in the respective cohorts who have both high-quality data of gut microbiome and genotype data, resulting in 16,017 participants across 4 Swedish cohorts. The need for larger size for a microbiome GWAS is a recognized issue in the field (Sanna et al, Nat Genet 2022, 54:100-106). However it is challenging to combine data from multiple cohorts to increase power due to biological and technical variation, including in metagenomic data processing, among cohorts. This study is the largest multi-cohort analysis with microbiome data processed with a harmonized bioinformatics pipeline in each of the cohorts. The sample size of each cohort included is outlined below.
SCAPIS: For this analysis, 8,733 participants of European ancestry from the Malmö and Uppsala sites data were included.
SIMPLER-Västmanland and SIMPLER-Uppsala: SIMPLER-V includes 4,515 COSM and SMC participants from Västmanland examined between 2010 and 2019. SIMPLER-U includes 981 women from the county of Uppsala, examined between 2003 and 2009 (no stool collected) and re-examined between 2015 and 2019 (stool collected).
MOS: The current analysis included 1,788 participants.

Data exclusions

For genotyping data, samples from individuals of non-European ancestry, failure in sex check, excess heterozygosity, and other quality control criteria including Hardy-Weinberg equilibrium, and minor allele frequency or count, were excluded. For gut microbiome data, only data that passed quality control was included.

Sensitivity analysis excluding individuals with antibiotic use in the past 6 months or self-reported inflammatory bowel disease;
exclusion of individuals who used antibiotics in the last six months or self-reported inflammatory bowel disease did not impact the genome-wide significant associations;
exclusion of persons in the same household resulting in only one person per household from SIMPLER and MOS did not impact results from SIMPLER and MOS cohorts;
excluding related participants resulting in only one participant from each related pair (meaning, no more related participant up to 3rd degree) did not affect the genome-wide findings;
MOS: Participants who were also part of the SCAPIS cohort were excluded from the MOS data.

Replication

Replication was conducted in the large Norwegian HUNT cohort of 12,652 individuals. We also used published summary statistics from two previous studies in FINRISK (n=5,959) and Dutch Microbiome Project (n=7,738) to validate the present findings. Best matching species were identified and our results were consistent with all 7 available associations in FINRISK, and 2 out of 4 in the Dutch cohort. The study in FINRISK used an earlier GTDB version (R89) for taxonomic annotations compared to our study (R214) while the Dutch study annotated their taxa by using MetaPhlAn2, which uses NCBI nomenclature.

Randomization

This is a population-cohort study and not an intervention study. Thus randomization is not applicable.

Blinding

This is a population-cohort study and not an intervention study. Thus blinding is not applicable.

Reporting for specific materials, systems and methods

We require information from authors about some types of materials, experimental systems and methods used in many studies. Here, indicate whether each material, system or method listed is relevant to your study. If you are not sure if a list item applies to your research, read the appropriate section before selecting a response.

Materials & experimental systems

n/a	Involved in the study
☒	☐ Antibodies
☒	☐ Eukaryotic cell lines
☒	☐ Palaeontology and archaeology
☒	☐ Animals and other organisms
☒	☐ Clinical data
☒	☐ Dual use research of concern

Methods

n/a	Involved in the study
☒	☐ ChIP-seq
☒	☐ Flow cytometry
☒	☐ MRI-based neuroimaging
